# Parents’ and Guardians’ Intentions to Vaccinate Children against COVID-19

**DOI:** 10.3390/vaccines10030361

**Published:** 2022-02-25

**Authors:** Pearl A. McElfish, Don E. Willis, Sumit K. Shah, Sharon Reece, Jennifer A. Andersen, Mario Schootman, Gloria Richard-Davis, James P. Selig, T. Scott Warmack

**Affiliations:** 1 College of Medicine, University of Arkansas for Medical Sciences Northwest, 1125 N. College Ave., Fayetteville, AR 72703, USA; dewillis@uams.edu (D.E.W.); screece@uams.edu (S.R.); jaandersen@uams.edu (J.A.A.); mschootman@uams.edu (M.S.); 2 Office of Community Health and Research, University of Arkansas for Medical Sciences Northwest, 1125 N. College Ave., Fayetteville, AR 72703, USA; sshah3@uams.edu; 3 College of Medicine, University of Arkansas Medical Sciences, 4301 W. Markham St., Little Rock, AR 72205, USA; garicharddavis@uams.edu; 4 Fay W. Boozman College of Public Health, University of Arkansas for Medical Sciences Northwest, 1125 N. College Ave., Fayetteville, AR 72703, USA; jpselig@uams.edu; 5 College of Pharmacy, University of Arkansas for Medical Sciences Northwest, 1125 N. College Ave., Fayetteville, AR 72703, USA; warmackthomass@uams.edu

**Keywords:** COVID-19, parent/guardian, child, vaccine hesitancy

## Abstract

A cross-sectional survey design was used to assess Arkansas parents’/guardians’ intentions to vaccinate their child against COVID-19. Parents/guardians whose oldest child was age 0–11 years (*n* = 171) or 12–17 years (*n* = 198) were recruited between 12 July and 30 July 2021 through random digit dialing. Among parents/guardians with an age-eligible child, age 12–17, 19% reported their child had been vaccinated, and 34% reported they would have their child vaccinated right away. Among parents/guardians with a child aged 0–11, 33% of parents/guardians reported they would have their child vaccinated right away. Twenty-eight percent (28%) of parents/guardians whose oldest child was 12–17 and 26% of parents/guardians whose oldest child was 0–11 reported they would only have their child vaccinated if their school required it; otherwise, they would definitely not vaccinate them. For both groups, parents’/guardians’ education, COVID-19 vaccination status, and COVID-19 vaccine hesitancy were significantly associated with intentions to vaccinate their child. More than a third of parents/guardians whose child was eligible for vaccination at the time of the survey reported they intended to have them vaccinated right away; however, they had not vaccinated their child more than two months after approval. This finding raises questions about the remaining barriers constraining some parents/guardians from vaccinating their child.

## 1. Introduction

COVID-19 was the third leading cause of death in the United States (US) in November 2021 and the leading cause of death in October 2021 for people aged 45–54 years [1]. Vaccination is critical to reduce the risk of hospitalization and death among adults and children [2]. The Emergency Use Authorization (EUA) for the COVID-19 vaccine was expanded in the US to include children age 12 and older on 10 May 2021 [3] and expanded to include children age 5 and older on 29 October 2021 [4]. Although children are less likely to have severe symptoms [5,6], children contribute to community transmission [5,7]. Furthermore, there are racial and ethnic disparities among children in COVID-19 related infections, hospitalizations, and deaths, and early data suggest children of a certain race/ethnicity who are eligible for the COVID-19 vaccine may be less likely to be vaccinated [8].

In the US, COVID-19 vaccination is widely available at clinics, pharmacies, public health departments, and vaccine outreach events. There is no charge to the patient/family for the COVID-19 vaccine regardless of insurance status. Arkansas is a rural, southern US state with low vaccination uptake of the COVID-19 vaccine, making it an important population to study. As of December 2021, children make up 18% of all COVID-19 cases in the state, putting the total count of child cases just under 100,000 [9]. Parental/guardian consent is required in most US states [10] for a child under the age of 18 to receive the COVID-19 vaccination, making it important to understand parents’/guardians’ intentions. Yet, few studies in the US have been conducted to understand parent/guardian intent to have their child receive the COVID-19 vaccination. Studies conducted in other countries have demonstrated sociodemographic characteristics, such as the age of parents, age of children, education, and income, are associated with willingness/hesitancy to have their children vaccinated [11,12,13,14]. In addition, several studies have demonstrated a parent’s vaccination status is strongly associated with willingness/hesitancy to have their children vaccinated [11,15].

The available studies in the US show race and ethnicity, gender, age, vaccine status, education, parents’ COVID-19 infection, and COVID-19 death exposure are associated with parents’/guardians’ intentions to vaccinate their child against COVID-19 [16,17,18]. Many published studies in the US used nonrandom samples [19,20,21], and very few peer-reviewed, representative samples surveyed parents/guardians after the EUA authorization for children 12–17. This leaves a significant gap in the literature. 

This study aimed to assess child vaccination and parent/guardian intentions to vaccinate their oldest child against COVID-19 and how intentions differed across parents’/guardians’ sociodemographic characteristics (gender, race, and education), health insurance status, vaccination status, parents’ COVID-19 infection, COVID-19 death exposure, perceived vaccination status of peers, and COVID-19 vaccine hesitancy.

## 2. Methods

### 2.1. Procedures

#### 2.1.1. Eligibility, Recruitment, and Consent

Arkansas residents were recruited between July 12 and July 30, 2021 using random digit dialing of telephone landlines and cell phones, with oversampling of African American/Black and Hispanic residents. Interviewers invited eligible participants to participate in the study and complete the survey. Interviewers explained: (1) the estimated time (10 min); (2) potential risks and benefits; (3) the voluntary nature of participation; and (4) confidentiality of responses. Consent was indicated by agreeing to participate in the survey. The survey had a cooperation rate of 20%. Study procedures were approved by an institutional review board for the protection of human subjects at the University of Arkansas for Medical Sciences (IRB # 262907).

#### 2.1.2. Data Collection and Analytical Sample

Telephone interviews were conducted by trained interviewers in English or Spanish depending on the respondent’s language of choice. The average length of the interview was 11 min. Data were collected from 1500 participants. The two analytical samples for this study consist of all respondents who indicated they were a parent or guardian whose oldest child was age 11 or younger (*n* = 171) or between the ages of 12 and 17 (*n* = 198). These samples were analyzed separately because parents and guardians were presented with different questions about child vaccination depending on the eligibility of their oldest child for COVID-19 vaccination at the time of the survey.

### 2.2. Measures

#### 2.2.1. Child Vaccination Intentions

Parents/guardians whose oldest child was not yet eligible for a vaccine were asked, “Once there is a COVID-19 vaccine authorized for your child’s age group, do you think you will…”, and parents/guardians whose oldest child was age 12–17 were asked, “Regarding a COVID-19 vaccine for your child, do you think you will…”. Response options for both questions included “get them vaccinated right away,” “wait a while to see how it is working,” “only get your child vaccinated if their school requires it,” “definitely not get them vaccinated,” and “child is already vaccinated.” Children under 12 were not eligible for vaccination at the time of this survey; therefore, no parents/guardians whose oldest child was age 11 or younger reported their child was already vaccinated.

#### 2.2.2. Sociodemographic Characteristics

Sociodemographic information included age, gender, race/ethnicity, and education. Data are presented for White, African American/Black, Multiracial/Other, and Hispanic groups. Multiracial and other racial groups (i.e., American Indian or Alaska Native, Asian, Pacific Islander, or Other) were combined due to a low frequency of responses. Any person who selected multiple racial identities was considered multiracial, and any person who reported they were of Hispanic, Latino/a, or Spanish origin were categorized as Hispanic/Latino. Individuals who were both multiracial and Hispanic/Latino were categorized as Hispanic/Latino. Respondents were asked for the highest degree or level of school they had completed and could answer with less than high school, some high school, high school graduate or equivalent (e.g., GED), some college but no degree, associate degree, bachelor’s degree, or graduate degree. Respondents with the responses less than high school and only some high school were combined with high school graduates due to low frequency.

#### 2.2.3. Health Insurance

Respondents could answer “yes” or “no” to the question, “Do you have any kind of health care coverage, including health insurance, prepaid plans such as HMOs, government plans such as Medicare, or Indian Health Service?” 

#### 2.2.4. COVID-19 Vaccination 

Respondents could answer “yes” or “no” to the question, “Have you received a COVID-19 vaccine?”

#### 2.2.5. Parents’ COVID-19 Infection 

Prior infection with COVID-19 was measured by asking, “Have you tested positive for or suspect that you have had COVID-19?” Response options included “Yes—I tested positive for COVID-19,” “Yes—I suspect that I had COVID-19,” and “No, I do not believe I have had COVID-19.” We dichotomized this measure by combining the affirmative responses and the negative response.

#### 2.2.6. COVID-19 Death Exposure

To measure COVID-19 death exposure, we asked, “Has a close friend or relative died of COVID-19?” Response options were “yes” and “no”.

#### 2.2.7. Perceived Vaccination Status of Peers

To measure the perceived social norms of vaccination, respondents were asked, “Of the people close to you, what proportion do you think have received the COVID-19 vaccine?” Response options included “very few”, “some, but not many”, “many”, and “nearly all”.

#### 2.2.8. COVID-19 Vaccine Hesitancy 

To measure COVID-19 vaccine hesitancy, respondents were asked, “Thinking specifically about the COVID-19 vaccines, how hesitant were/are you about getting vaccinated?” Response options included “not at all hesitant,” “a little hesitant,” “somewhat hesitant,” and “very hesitant.” This question was asked regardless of whether they had been vaccinated already or not, as receipt of the COVID-19 vaccine should not be conflated with the absence of hesitancy [22].

### 2.3. Analyses

Data were analyzed using STATA 15.1 SE. Arkansas 2019 census estimates for age, race, and sex were used to generate weights using raking ratio estimation. We present results for parents/guardians whose oldest child was age 11 or younger (*n* = 171) or between ages 12 and 17 (*n* = 198), including weighted descriptive statistics and bivariate analyses including Pearson χ^2^ statistics with Rao and Scott second-order correction [23,24]. We present only descriptive statistics (mean) for age across the five possible responses of the dependent variable. Missing data comprised less than 5% of the total and were handled through pairwise deletion.

## 3. Results

The weighted descriptive statistics for the sample are presented in Table 1. The mean age of parents/guardians whose oldest child was 11 or younger was 36 years and 44 years for parent/guardians whose oldest child was between ages 12 and 17. Closely matching the general racial composition of the state of Arkansas, most parents/guardians whose oldest child was age 0–11 identified as White (70%), followed by Black (15%), Hispanic (11%), and Multiracial/Other (4%). Results were similar for parents/guardians whose oldest child was age 12–17—most identified as White (72%), followed by Black (13%), Hispanic (9%), and Multiracial/Other (6%). The most prevalent education level for both subsamples was a high school degree or less, which made up 30% of parents/guardians whose oldest child was age 0–11 and 23% of parents/guardians whose oldest child was age 12–17.

More than 80% of respondents in both samples had health insurance, and more than 60% had received at least one dose of a COVID-19 vaccine themselves. Among parents/guardians whose oldest child was age 11 or younger, 21% had experienced the death of a close friend or relative due to COVID-19. Among parents/guardians whose oldest child was age 12–17, 31% experienced the death of a close friend or relative due to COVID-19. More than half of respondents in both subsamples reported many or nearly all people close to them were vaccinated against COVID-19.

Among parents/guardians whose oldest child was age 11 or younger, a third were planning to vaccinate their child right away, and 41% were waiting to see. Almost 10% planned to vaccinate their child only if required, and 17% reported they would definitely not vaccinate their child against COVID-19. Among parents/guardians whose oldest child was between 12 and 17 years old, 19% reported their child was already vaccinated, a third were planning to vaccinate their child right away, and a fifth were waiting to see. Almost 10% planned to vaccinate their child only if required, and almost a fifth reported they would definitely not vaccinate their child against COVID-19. The prevalence of responses for both parent/guardian samples was similar, with a higher proportion of respondents whose oldest child was age 11 or younger responding they would wait and see; however, these responses are not directly comparable given parents whose children were eligible for vaccination had the additional option of reporting their children were already vaccinated. 

The bivariate analyses for parents’/guardians’ intentions to vaccinate their oldest child who was aged 11 or younger is presented in Table 2. Significant differences in vaccination plans for children were found across education (F = 2.45; *p* = 0.006). Respondents with a graduate or professional degree, followed by those with a bachelor’s degree, had the highest prevalence out of those reporting they would have their child vaccinated right away and the lowest prevalence out of those reporting they would refuse. Nearly a third (30%) of parents/guardians with a high school degree or less stated they would not have their child vaccinated against COVID-19.

Significant differences were observed across health insurance (F = 5.39; *p* = 0.001). The most prevalent response among insured parents/guardians was to have their child vaccinated right away, while the most prevalent response among those with no insurance was to wait and see. The prevalence of planning to refuse to vaccinate a child was higher among insured parents/guardians than those without insurance. 

Significant differences in vaccination plans for children were found across those who had and had not received the COVID-19 vaccination (F = 19.95; *p* < 0.001). Over half of vaccinated parents/guardians planned to have their child vaccinated right away, compared to just two percent of unvaccinated parents/guardians. Significant differences in vaccination plans for children were found across the perceived vaccination status among the parents’/guardians’ peers (F = 4.19; *p* < 0.001). Reports of plans to have children vaccinated right away were less prevalent for parents/guardians who perceived fewer people close to them to be vaccinated, and the prevalence of plans to refuse the vaccine was highest among parents/guardians who perceived fewer people close to them to be vaccinated. 

Significant differences in vaccination plans for children were found across COVID-19 vaccine hesitancy (F = 7.66; *p* < 0.001). Reports of plans to have children vaccinated right away were less prevalent for parents/guardians who reported higher levels of COVID-19 vaccine hesitancy, and the prevalence of plans to refuse the vaccine was highest among parents/guardians who reported higher levels of COVID-19 vaccine hesitancy.

Bivariate analyses are presented for parents’/guardians’ intentions to vaccinate their oldest child, who is between 12 and 17, in Table 3. Parent/guardian age was not associated with vaccination plans for their child (F_4, 176_ = 2.21, *p* = 0.070). Significant differences in vaccination plans for children were found across education levels (F_15, 2882._ = 1.69; *p* = 0.043). Parents/guardians with bachelor’s or graduate degrees had the highest prevalence of children who were already vaccinated or children they planned to have vaccinated right away. Plans to refuse or only have their child vaccinated if required were most prevalent among parents/guardians with a high school degree or less. 

Significant differences in vaccination plans for children were found across COVID-19 vaccination status (F_3, 701_ = 22.76; *p* < 0.001). Vaccination among children or plans to vaccinate them right away were most prevalent among parents/guardians who had received at least one dose of the COVID-19 vaccine themselves. Significant differences in vaccination plans for children were found across the perceived vaccination status among the parents’/guardians’ peers (F_11, 2142_; *p* < 0.01). Vaccination among children or plans to vaccinate them right away were most prevalent among parents/guardians who believed a greater number of people close to them were vaccinated. 

Significant differences in vaccination plans for children were found across COVID-19 vaccine hesitancy (F_10, 1928_ = 7.82; *p* < 0.001). Vaccination among children or plans to vaccinate them right away were most prevalent among parents/guardians who were less hesitant about COVID-19 vaccines.

## 4. Discussion

The survey was conducted in the final weeks of July 2021, and the expanded COVID-19 vaccination EUA for children age 12–17 was approved by the FDA in the US two months earlier on 10 May 2021. At the time of the survey, only 19% of parents/guardians whose oldest child was age-eligible reported they had been vaccinated. Interestingly, 34% reported they would have their child vaccinated right away but had not yet realized this. Some studies have shown significant logistical barriers to vaccination that may constrain vaccination uptake, especially among communities of color [16,25]. Future mixed-methods research should examine why those who are not hesitant delay vaccination for their child, as additional research is needed to help those parents overcome possible logistical barriers. 

More than a quarter of parents/guardians for both age groups reported they would only have their child vaccinated if their school required it; otherwise, they would definitely not have them vaccinated. Although the proportion is less than reported in prior studies [16,18], this could have significant implications for ending the COVID-19 pandemic because population immunity is estimated to require a vaccination rate close to 90% of the US population [26,27]. 

For both age groups, parents’/guardians’ education, COVID-19 vaccination status, and COVID-19 vaccine hesitancy were significantly associated with intentions to vaccinate their child. This is consistent with the limited number of prior studies conducted in the US [16,17,18] and in other countries [11,15]. For both age groups, parents’/guardians’ perceived peer vaccination status was significantly associated with intent to have their child vaccinated against COVID-19. This is one of the very few studies to examine social norms on parents’/guardians’ intent to vaccinate their child. Although prior studies have considered the influence of social norms and the perceived vaccination status of peers, most have been focused on flu vaccination [28,29]. The only other study to our knowledge to assess the relationship with intentions to vaccinate children against COVID-19 was a direct survey of adolescents themselves, rather than parents/guardians [30]. 

There were not significant associations between intentions to vaccinate children against COVID-19 and race/ethnicity. These findings are encouraging and in contrast with some prior findings, which have shown differences based on race/ethnicity [17,18]. However, prior studies have documented additional barriers to vaccination among communities of color [16], including transportation, health care access, and time off work. There were also no significant associations between child vaccination intent and gender, parents’ COVID-19 infection, or COVID-19 death exposure for either subsample of parents/guardians. The associations based on gender have been mixed, and the findings are in contrast to studies in the US [17,18] and other countries [11], which have shown females report greater hesitancy to have their child receive the COVID-19 vaccine. To our knowledge, only one other study has examined associations between parents’/guardians’ intentions to have their child vaccinated against COVID-19 and parents’/guardians’ prior COVID-19 infection or COVID-19 death exposure in the US [17]. Our findings differ, however, as we found no significant associations with these variables.

### Limitations

This is one of the first studies to examine parents’/guardians’ intentions to have their child vaccinated against COVID-19 using a diverse, random sample in a southern, rural US state, shortly after the EUA of the COVID-19 vaccine for children 12–17 years of age. This is the first study to examine parents of those 12–17 separately from those 0–11. The study is limited by a small sample size, and the study used cross-sectional data, which do not allow causal claims. Moreover, the survey cooperation rate was 20%, which is lower than we would prefer but higher than that of other well-respected survey research centers [31]. The study focused on parents’/guardians’ intent to have their child receive the COVID-19 vaccination, which is appropriate because most states require parental consent for child vaccination. However, youth have opinions and views of their own regarding vaccines [32]. Some US states do not require parental consent for vaccinations; therefore, vaccination among those under age 18 may not depend entirely on parent/guardian intentions. Additional research is needed to understand youth’s COVID-19 vaccine intent, behaviors, and attitudes, especially in states where parental consent is not required.

## 5. Conclusions

This study has significant implications for COVID-19 vaccine implementation among children. More than a third of parents/guardians who had children eligible to be vaccinated reported they intended to have them vaccinated right away; however, they had not had their child vaccinated more than two months after approval. This finding raises additional questions about the remaining barriers that may still constrain some parents/guardians from having their child vaccinated even though they have intentions to do so right away. Public health practitioners must ensure convenient access to vaccination to ensure those who intend to have their child vaccinated are able to do so. For those who plan to wait and see, it is critical that public health practitioners continue to provide information about the safety and efficacy of the vaccination. The fact that almost 25% of respondents, regardless of their child’s age group, stated they had no plans to vaccinate their child, or will only do so if their school requires it, could significantly affect efforts to end the COVID-19 pandemic. 

## Figures and Tables

**Table 1:** Weighted descriptive statistics of parents/guardians of a child age 0–11 and 12–17.

	Children 0–11	Children 12–17
	Column %	Column %
**Age (mean)**	36	44
	(*n* = 168)	(*n* = 189)
**Gender**		
	(*n* = 171)	(*n* = 198)
Men	52	47
Women	48	53
**Race**		
	(*n* = 170)	(*n* = 198)
White	70	72
Black	15	13
Multiracial/Other	4	6
Hispanic	11	9
**Education**		
	(*n* = 170)	(*n* = 197)
High school or less	30	23
Some college	25	19
Associate degree	10	17
Bachelor’s degree	27	21
Graduate degree	8	19
**Health Insurance**		
	(*n* = 170)	(*n* = 197)
Not insured	16	11
Insured	84	89
**COVID-19 Vaccination**		
	(*n* = 171)	(*n* = 197)
At least one dose	61	66
No doses	39	34
**Parents’ COVID-19 Infection**		
	(*n* = 170)	(*n* = 196)
Yes	30	29
No	70	71
**COVID-19 Death Exposure**		
	(*n* = 168)	(*n* = 196)
Yes	21	31
No	79	69
**Perceived Peer Vaccination Status**		
	(*n* = 171)	(*n* = 198)
Very few	17	12
Some but not many	24	29
Many	33	31
Nearly all	26	28
**COVID-19 Vaccine Hesitancy**		
	(*n* = 171)	(*n* = 198)
Not at all hesitant	43	50
A little hesitant	15	16
Somewhat hesitant	13	6
Very hesitant	29	28
**Child Vaccination Intentions**		
	(*n* = 167)	(*n* = 189)
Already vaccinated	--	19
Right away	33	34
Wait and see	41	20
Only if required	9	9
Definitely not	17	19

**Note:** Bold font does not indicate significance and is for organizational purpose only.

**Table 2:** Weighted bivariate relationships—parents of a child age 0–11 years.

	Right Away	Wait and See	Only If Required	Definitely Not	*p*
	**Row %**				
**^a^ Age (mean)**	37	34	32	37	0.105
(*n* = 164)					
**Gender**					0.765
(*n* = 167)					
Men	32	43	7	18	
Women	34	38	12	16	
**Race**					0.263
(*n* = 167)					
White	35	38	8	19	
Black	36	43	14	7	
Multiracial/Other	9	44	8	39	
Hispanic	26	55	9	10	
**Education**					0.006
(*n* = 166)					
High school or less	20	37	12	30	
Some college	29	52	6	13	
Associate degree	4	52	26	18	
Bachelor’s degree	52	33	5	11	
Graduate degree	64	28	4	3	
**Health Insurance**					0.001
(*n* = 166)					
Not insured	7	52	29	11	
Insured	38	37	6	19	
**COVID-19 Vaccination**					<0.001
(*n* = 167)					
At least one dose	53	41	6	1	
No doses	2	40	15	43	
**Parents’ COVID-19 Infection**					0.354
(*n* = 166)					
Yes	24	41	9	26	
No	36	41	10	13	
**COVID-19 Death Exposure**					0.235
(*n* = 164)					
Yes	24	58	6	12	
No	35	35	10	19	
**Perceived Peer Vaccination Status**					<0.001
(*n* = 167)					
Very few	13	35	2	49	
Some but not many	19	42	17	22	
Many	33	43	13	11	
Nearly all	59	39	2	0	
**COVID-19 Vaccine Hesitancy**					<0.001
(*n* = 167)					
Not at all hesitant	57	33	5	5	
A little hesitant	47	49	4	0	
Somewhat hesitant	8	69	10	14	
Very hesitant	1	35	18	46	

^a^ Adjusted Wald test. **Note:** Bold font does not indicate significance and is for organizational purposes only.

**Table 3:** Weighted bivariate relationships—parents of a child age 12–17 years.

	Already Vaccinated	Right Away	Wait and See	Only If Required	Definitely Not	*p*
	**Row %**					
**^a^ Age (mean)**	45	42	47	43	47	0.070
(*n* = 180)						
**Gender**						0.224
(*n* = 189)						
Men	15	27	24	10	24	
Women	22	40	16	8	13	
**Race**						0.942
(*n* = 189)						
White	18	33	21	8	19	
Black	17	38	13	16	16	
Multiracial/Other	26	27	12	9	27	
Hispanic	19	36	21	10	13	
**Education**						0.043
(*n* = 188)						
High school or less	9	40	13	8	30	
Some college	13	18	23	20	26	
Associate degree	10	35	31	9	15	
Bachelor’s degree	26	28	21	5	20	
Graduate degree	34	47	13	5	0	
**Health Insurance**						0.463
(*n* = 188)						
Not insured	7	40	16	7	30	
Insured	20	32	20	9	18	
**COVID-19 Vaccination**						<0.001
(*n* = 189)						
At least one dose	27	48	18	6	1	
No doses	3	6	23	16	53	
**Parents’ COVID-19 infection**						0.055
(*n* = 187)						
Yes	23	37	19	5	17	
No	10	26	22	18	24	
**COVID-19 Death Exposure**						0.095
(*n* = 187)						
Yes	23	41	11	15	10	
No	16	32	24	7	22	
**Perceived Peer Vaccination Status**						0.006
(*n* = 189)						
Very few	9	34	14	13	30	
Some but not many	20	19	6	19	36	
Many	21	35	28	5	12	
Nearly all	19	48	27	2	4	
**COVID-19 Vaccine Hesitancy**						<0.001
(*n* = 189)						
Not at all hesitant	29	52	9	6	4	
A little hesitant	14	30	50	5	1	
Somewhat hesitant	7	5	42	0	46	
Very hesitant	5	10	17	19	49	

^a^ Only descriptive statistics reported for age. **Note:** Bold font does not indicate significance and is for organizational purposes only.

## Data Availability

The deidentified data underlying the results presented in this study may be made available upon request from the corresponding author, Pearl A. McElfish, at pamcelfish@uams.edu. The data are not publicly available in accordance with funding requirements and participant privacy.
